# Instruments assessing mobility of children and adolescents with autism spectrum disorder: A systematic review and decision map

**DOI:** 10.1111/dmcn.70136

**Published:** 2025-12-29

**Authors:** Arthur Felipe Barroso de Lima, Amanda Cristina Fernandes, Amanda Alves Rodrigues Soares, Hércules Ribeiro Leite, Ricardo Rodrigues de Sousa Junior

**Affiliations:** ^1^ Undergraduate Program in Physical Therapy Universidade Federal de Minas Gerais Belo Horizonte Brazil; ^2^ Graduate Program in Rehabilitation Sciences, School of Physical Education, Physical Therapy and Occupational Therapy Universidade Federal de Minas Gerais Belo Horizonte Brazil

## Abstract

**Aim:**

To identify the standardized instruments used to assess mobility aspects in children and adolescents with autism spectrum disorder (ASD), analyse the quality of their psychometric properties and their level of evidence, and develop a clinical decision map for these instruments.

**Method:**

Articles were screened and study characteristics were extracted. The methodological quality of the selected studies was analysed using the COSMIN Risk of Bias checklist. The quality of evidence for each measurement property was defined using a modified version of the Grading of Recommendations Assessment, Development and Evaluation (GRADE) system.

**Results:**

Eleven instruments were analysed in 11 studies. Of these instruments, three are directed towards performance assessment and eight towards capacity assessment. The selected studies evaluated the psychometric properties of Vineland Adaptive Behavior Scales, Gross Motor Assessment of Children and Adolescents with Autism Spectrum Disorder, Ignite Challenge, Pediatric Evaluation of Disability Inventory Computer Adaptive Test, Miller Function and Participation Scales, Peabody Developmental Motor Scales, Second Edition, Test of Gross Motor Development, Second and Third Editions, Timed Up and Go, Developmental Coordination Disorder Questionnaire, and Movement Assessment Battery for Children, Second Edition. Of these instruments, nine were developed for the evaluation of typically developing children and children with disabilities, and have been validated for the population with ASD (81.8%). The other two instruments (18.2%) were specifically developed for the evaluation of the population with ASD.

**Interpretation:**

Most (56.51%) of the measurement properties of the instruments demonstrated low or very low evidence because of risk of bias and imprecision, reinforcing the importance of further studies to strengthen the validity and applicability of these assessments.

## Graphical abstract

Portuguese translation of this Systematic Review is available in the online issue.

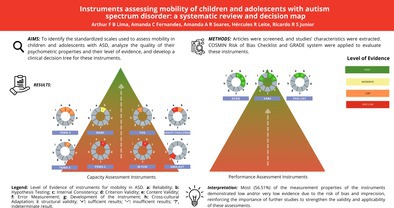

**Abbreviations**
ASD: autism spectrum disorder
DCDQ: Developmental Coordination Disorder Questionnaire
GMA‐AUT: Gross Motor Assessment of Children and Adolescents with Autism Spectrum Disorder
ICF: International Classification of Functioning, Disability and Health
MABC‐2: Movement Assessment Battery for Children, Second Edition
M‐FUN: Miller Function and Participation Scales
PDMS‐2: Peabody Developmental Motor Scales, Second Edition
PEDI‐CAT: Pediatric Evaluation of Disability Inventory Computer Adaptive Test
TGMD‐2: Test of Gross Motor Development, Second Edition
TGMD‐3: Test of Gross Motor Development, Third Edition
TUG: Timed Up and Go
VABS: Vineland Adaptive Behavior Scales

**What this paper adds**

- Robust studies confirming the evidence level of these instruments for autism spectrum disorder (ASD) are still lacking.
- A decision map based on the evidence directs the selection of appropriate mobility aspect assessments in ASD.
- Future analysis of the measurement properties of instruments is needed.

Autism spectrum disorder (ASD) is primarily characterized by deficits in social interaction, communication, and the presence of restricted and repetitive behaviours, interests, or activities.^1^ In recent years, the prevalence of ASD has increased significantly. Recent data indicates that 1 in 36 children in the USA has been diagnosed with ASD.^2^ The causes of the condition are multifactorial, involving environmental and genetic factors, which contribute to the variability in the clinical presentation and functioning of these individuals.^3^

According to the International Classification of Functioning, Disability and Health (ICF), functioning encompasses body functions and structures, activities, and participation, as well as contextual factors (personal and environmental).^4^ Individuals with ASD may exhibit several impairments that hinder their functioning. These impairments frequently result in limitations across different life areas, especially mobility, which tends to be underassessed in this population.^5^, ^6^ According to the ICF, mobility is a fundamental motor aspect within the activity domain of the ICF, involving the ability to move in the environment (e.g. changing and maintaining body position), transporting or moving objects (e.g. carrying or handling), and locomotion (e.g. walking and moving around).^4^ Recent studies showed a high prevalence of limitation in these mobility aspects in children and adolescents with ASD.^6^, ^7^, ^8^, ^9^

Evidence related to ASD highlights mobility limitations in fine motor, balance and coordination, locomotor, and object control skills.^6^ These aspects may occur because of deficits in motor praxis, hypotonia, and low endurance found in this group of children and adolescents.^6^, ^7^, ^8^, ^9^ These limitations together with social limitations may hinder the participation of children and adolescents in physical activity; they present lower levels of physical activity compared to their typically developing peers.^10^, ^11^ In addition, mobility limitations may act as barriers to full participation in other life areas of children and adolescents with ASD, affecting their autonomy and social and family integration.^12^, ^13^, ^14^

Standardized tools for assessing social interaction, communication, and restricted, repetitive behaviours in ASD are well established; however, tools for evaluating mobility in this population remain limited.^15^, ^16^ Several instruments exist to assess capacity (task execution in a standardized environment)^4^ and performance (task execution in the child's everyday environment) of mobility in children with neurodevelopmental disorders.^4^ Capacity and performance are distinct but complementary aspects of mobility in children with ASD, for example, capacity reflects how accurately a child performs a motor task, whereas performance reflects whether assistance is needed to complete the motor task in daily life. However, applying these tools to children and adolescents with ASD can be complex. Some tests may not capture relevant motor difficulties.^17^ Furthermore, restricted interests, limited attention, and difficulties engaging with the assessor can reduce patient collaboration, while cognitive impairments in those requiring higher levels of support may hinder comprehension of instructions. In addition, many movement specialists, such as physical therapists, report a lack of expertise, confidence, and formal training when assessing mobility in this population.^18^ A stronger understanding of standardized mobility assessments may enhance the clinical skills of therapists and help them address these challenges more effectively.

Recently, two systematic reviews investigated general motor assessment tools in children and adolescents with ASD.^17^, ^19^ These studies presented instruments mostly for children with a high risk of autism, without including adolescents, with a lack of focus on mobility aspects. In addition, the quality of the measurement properties of these tools was not evaluated.^17^, ^19^ This may pose a limitation for clinical practitioners in selecting the most appropriate instruments based on their construct and the level of evidence of their measurement properties.

Thus, analysing the available tools with regard to the evaluation of mobility aspects and their measurement properties can provide evidence‐based recommendations, aiding clinicians in selecting standardized and suitable assessment scales for evaluating children and adolescents with ASD. Accordingly, the present study aimed to identify the standardized tools used to assess mobility aspects in children and adolescents with ASD. Additionally, it sought to analyse the quality of their psychometric properties and their level of evidence, and develop a clinical decision map to facilitate clinical reasoning during the evaluation process of this population.

## METHOD

This systematic review was conducted based on the Preferred Reporting Items for Systematic reviews and Meta‐Analyses (PRISMA)‐COnsensus‐based Standards for the selection of health Measurement INstruments (COSMIN) criteria for outcome measurement instruments and followed the COSMIN guidelines for conducting systematic reviews of outcome measurements.^20^, ^21^ This review was registered in the International Prospective Register of Systematic Reviews (PROSPERO) under registration no. CRD42023485879.

### Search strategy and selection

A search was conducted in the PubMed, Web of Science, Embase, PsycINFO, Scopus, and SciELO databases in October 2023. The search was updated in August 2025 to capture new studies. The selection process was executed by two independent reviewers. The search strategy was developed using keywords related to the PICO framework. By following the PICO framework (population, indicator, comparison, and outcome) this study answered the question: Among children and adolescents with ASD (P), what standardized tools (I) are used to assess mobility aspects, and what is the quality of their psychometric properties and level of evidence for this specific population (O)? Table S1 presents the keywords used for this study's search, which was based on the COSMIN search filter for systematic review of instruments.^22^

The reference lists of the selected studies and previous reviews on instruments for children with ASD^17^, ^19^ were manually searched to ensure that all potential studies evaluating the measurement properties of mobility assessments in children and adolescents with ASD were included. Additionally, complementary searches using the names of the assessments found in our main search and those mentioned in previous review studies were conducted. For these additional searches, we included as keywords the name of the instrument together with the COSMIN search filter.^22^

### Eligibility criteria

This study included psychometric studies that investigated one or more measurement properties of standardized assessment instruments, such as reliability and internal consistency, designed to evaluate mobility aspects in children and adolescents with ASD. Mobility was considered as defined in the ICF within the activity domain (i.e. changing and maintaining body position; carrying, moving, and handling objects; walking and moving; and moving around using transportation).^4^ Therefore, instruments evaluating other components were considered if they were primarily directed towards mobility aspects (>60% of the instrument items). These aspects included locomotion, transfers, object manipulation, and more for individuals with ASD aged 0 to 21 years. Studies included children and adolescents diagnosed with ASD in more than 50% of the sample. That also included children with other neurodevelopmental diagnoses, such as genetic syndromes, if there was information regarding ASD comorbidity or core autistic symptoms in the study's sample.

There were no restrictions on language or year of publication. The following studies were excluded: (1) those that did not include samples predominantly consisting of children and adolescents diagnosed with ASD (<50%); (2) abstracts; and (3) instruments targeting outcomes unrelated to mobility aspects.

### Data extraction and analysis

Two independent reviewers removed duplicates and screened titles and abstracts. Subsequently, the full texts of potentially eligible articles were assessed. Disagreements between reviewers were resolved by a third reviewer. Information regarding the characteristics of the studies and instruments (such as the outcomes assessed and application time) and population (such as sample size, age, and sex), country where the study was conducted, the evaluated measurement properties, and results (i.e. psychometric indexes) were extracted.

According to the COSMIN guidelines for conducting systematic reviews of instruments, data from the following measurement properties were extracted and investigated: (1) content validity; (2) structural validity (including factor structure); (3) internal consistency; (4) cross‐cultural validity and measurement of invariance; (5) reliability; (6) measurement of error; (7) criterion validity; (8) hypothesis testing for construct validity; (9) responsiveness; and (10) instrument development. The operational definitions of each of these measurement properties, and information on their quality criteria, can be found in the COSMIN guidelines.^20^

We also extracted information regarding the type of instrument according to the ICF descriptors of the activity domain, that is, performance and capacity. Performance is defined as the execution of a task in the habitual environment; capacity is the execution of a task in a standardized environment.^4^ In paediatric rehabilitation, performance is commonly evaluated using parental questionnaires regarding the child's ability in their own environment. Capacity is commonly evaluated using standardized objective motor assessments.

### Methodological quality of the studies

#### Risk of bias

The methodological quality of the selected studies was assessed using the COSMIN Risk of Bias checklist.^20^, ^23^, ^24^ Each measurement property evaluated in a study is rated on a 4‐point scale: very good; adequate; doubtful; or inadequate. The overall methodological quality for each property is determined by the lowest rating among the relevant criteria (i.e. the ‘worst score count’ principle). The COSMIN checklist includes distinct criteria for different measurement properties; therefore, a single study may receive different ratings for each property evaluated.^20^, ^23^, ^24^

#### Quality of measurement properties

The measurement properties reported in the studies were evaluated using the COSMIN quality criteria.^25^ Each property identified in the selected studies was classified into three categories: (+) sufficient, when the statistical psychometric indices met the COSMIN criteria; (−) insufficient, when they did not meet these criteria; or (?) indeterminate, in cases where the available information on psychometric indices was insufficient.

Risk of bias and the quality of measurement properties were assessed independently by two reviewers; if there was a discrepancy, a third reviewer was responsible for defining the measurement properties.

### Summary of results

The data from all studies that evaluated the same instrument and same measurement properties were synthesized to determine their overall methodological quality and general quality criteria for measurement properties, as recommended by Mokkink et al.^20^ The overall methodological quality of each instrument was established by summing the number of studies classified as very good, adequate, doubtful, and inadequate. The overall evaluation of each instrument's measurement properties was classified into four categories: (+) sufficient, when most properties (over 50%) were rated as +; (−) insufficient, when the majority (over 50%) were rated as −; (?) indeterminate, when most properties (over 50%) were rated as indeterminate (?); and (±) inconsistent, when the results showed notable discrepancies, such as an even distribution (e.g. 50% + and 50% −).

### Quality of evidence rating

The quality of evidence for each measurement property was defined using a modified version of the Grading of Recommendations Assessment, Development and Evaluation (GRADE) system proposed by Mokkink et al.^20^ The level of evidence was classified as high, moderate, low, or very low. In this system, four factors are considered to classify the quality of evidence for the psychometric properties of an instrument: risk of bias; inconsistency; imprecision; and indirectness. Overall, the GRADE approach begins with a high‐quality level of evidence, which can be downgraded stepwise to moderate, low, or very low, depending on the number and severity of limitations identified in these four domains.

The first factor, risk of bias, refers to the methodological quality of the studies included. The level of downgrading depends on the severity of methodological issues. Evidence is downgraded by one level (serious risk of bias) when studies are of doubtful quality or only one adequate‐quality study is available; by two levels (very serious risk of bias) when multiple studies show inadequate quality in the risk of bias assessment or only one study of doubtful quality exists; and by three levels (extremely serious risk of bias) when only one inadequate study is available.^20^

The second factor, inconsistency, concerns variability in study results for the same measurement property. If the results across studies are inconsistent (±) in the quality of the measurement properties, and no reasonable explanation can be provided (e.g. because of differences in populations or versions of the instrument), the evidence should be downgraded. It is important to note that when the measurement properties are classified as indeterminate (?) in the quality assessment, their level of evidence is not rated.

The third factor, imprecision, according to COSMIN standards, relates to the total sample size on which the overall rating is based. Small sample sizes reduce confidence in the stability of the findings. Evidence is downgraded by one level (serious imprecision) when the total sample size is below 100 participants, and by two levels (very serious imprecision) when it is below 50.^20^

Finally, indirectness reflects the degree to which the evidence is directly applicable to the population, construct, or context of interest. Evidence is considered indirect when the included studies were conducted in a different population (e.g. different populations other than ASD), when the instrument was used in a different context or purpose than intended, or when it assessed a different construct.^20^

The quality of evidence was interpreted as follows: high evidence, when we can confidently assume that the results of the instrument's psychometric properties are trustworthy; moderate evidence, when we can moderately assume that the results of the instrument's psychometric properties are trustworthy; low evidence, when our assumption of trustworthiness is limited and the results of the instrument's psychometric properties may differ from what is presented; and very low evidence, when we cannot assume the results are trustworthy and the results of the instrument's psychometric properties are likely to differ from what is presented, being inconsistent.^20^, ^23^

The final rating was determined through consensus between two reviewers, using a third reviewer in case of disagreement. These grading procedures (risk of bias, quality of measurement properties, summary of findings, and quality of evidence) strictly followed the COSMIN guidelines and the step process exemplified by Andrade et al.^20^, ^25^

## RESULTS

From the searches conducted, 6098 studies were identified and 11 were eligible. The flowchart that details the search and selection process is shown in Figure S1. Most of the studies included children and adolescents with a confirmed diagnosis of ASD. However, the methods considered for diagnosis, functional classification, and level of support were poorly reported in the studies that were selected.

The selected studies evaluated the psychometric properties of 11 instruments, namely, Vineland Adaptive Behavior Scales (VABS),^26^, ^27^ Gross Motor Assessment of Children and Adolescents with Autism Spectrum Disorder (GMA‐AUT),^28^ Ignite Challenge,^29^ Pediatric Evaluation of Disability Inventory Computer Adaptive Test (PEDI‐CAT),^30^ Miller Function and Participation Scales (M‐FUN),^31^ Peabody Developmental Motor Scales, Second Edition (PDMS‐2);^31^ Test of Gross Motor Development, Second and Third Editions (TGMD‐2 and TGMD‐3);^32^, ^33^ Timed Up and Go (TUG);^34^ Developmental Coordination Disorder Questionnaire (DCDQ),^35^ and Movement Assessment Battery for Children, Second Edition (MABC‐2).^36^ These studies were conducted in seven countries: Australia (*n* = 1),^30^ Belgium (*n* = 1),^35^ Brazil (*n* = 2),^28^, ^36^ Canada (*n* = 1),^29^ China (*n* = 1),^26^ Spain (*n* = 1),^34^ and the USA (*n* = 4).^27^, ^31^, ^32^, ^33^

### Instruments characteristics

Table 1 presents the main characteristics and information regarding the instruments. Of the instruments selected, nine were developed for the evaluation of typically developing children and validated for the population with ASD (81.8%). The other two instruments (18.2%) were specifically developed for the evaluation of the population with ASD, such as the GMA‐AUT and Ignite Challenge. Of the selected instruments, three are directed towards performance assessment and eight towards capacity assessment. Among the capacity instruments, there was variability of the mobility aspects evaluated, such as quality of execution of locomotor and object control skills (TGMD‐2 and TGMD‐3), capacity of execution of basic motor skills (GMA‐AUT), precision and time of execution of advanced motor skills (Ignite Challenge), and manual dexterity and balance skills (MABC‐2). In addition, there was variability in the age of the target population, which ranged from 0 to 21 years.

**Table 1:** Characteristics of the instruments.

Type of instrument	Instrument	Application time	Which professionals can administer this tool?	Age range	Specific for ASD	Certification and required materials	Outcomes
Performance assessment	PEDI‐CAT	15–30 minutes	Physical therapists, occupational therapists and professionals interested in assessing functional performance	0–21 years	Yes	No formal certification is required; purchasing the form is required	Functional performance evaluation in mobility activities
	DCDQ	20–30 minutes	Physical therapists and occupational therapists	5–15 years	No	No formal certification is required; purchase of materials is required	Presence of developmental coordination disorder
	VABS	Up to 60 minutes	Occupational therapists, psychologists, speech therapists	0–90 years	No	No formal certification is required; qualification/training is necessary for the application	Functional performance evaluation in mobility activities
Capacity assessment	GMA‐AUT	60 min	Physical therapists and occupational therapists	4–18 years	Yes	No formal certification is required; no material purchase is required	Observational assessment of gross motor function
	Ignite Challenge	30–45 minutes	Physical therapists and occupational therapists	6–14 years	Yes	Formal certification required; purchase of materials is required	Observational assessment of advanced motor skills
	M‐FUN	45–60 minutes	Physical therapists, occupational therapists, and professionals interested in assessing motor skills	2–7 years	No	No formal certification is required; purchase of materials is required	Observational assessment of gross motor function
	TGMD‐2	40–45 minutes	Physical therapists, occupational therapists, and professionals interested in assessing gross motor development	3–10 years	No	No formal certification required; purchase of materials is required	Observational assessment of advanced motor skills
	TGMD‐3	40–45 minutes	Physical therapists, occupational therapists, and professionals interested in assessing gross motor development	3–10 years	No	No formal certification is required; purchase of materials is required	Observational assessment of advanced motor skills
	PDMS‐2	60 minutes	Physical therapists and occupational therapists	0–6 years	No	No formal certification is required; purchase of materials is required	Observational assessment of gross motor function
	TUG	5 minutes	Physical therapists, occupational therapists, and professionals interested in assessing mobility aspects	Various age ranges	No	No formal certification is required; purchase of materials is required	Timed assessment of mobility (ability to stand, walk 3 metres, turn around a cone, and sit back)
	MABC‐2	20–40 minutes	Physical therapists, occupational therapists and professionals interested in assessing functional performance	3–16 years	No	No formal certification is required; purchase of materials and forms is required	Observational assessment of fine and gross motor function

Abbreviations: ASD, autism spectrum disorder; DCDQ, Developmental Coordination Questionnaire; GMA‐AUT, Gross Motor Assessment of Children and Adolescents with ASD; MABC‐2, Movement Assessment Battery for Children, Second Edition; M‐FUN, Miller Function and Participation Scales; PDMS‐2, Peabody Developmental Motor Scales, Second Edition; PEDI‐CAT, Pediatric Evaluation of Disability Inventory Computer Adaptive Test for Autism; TGMD‐2, Test of Gross Motor Development, Second Edition; TGMD‐3, Test of Gross Motor Development, Third Edition; TUG, Timed Up and Go; VABS, Vineland Adaptive Behavior Scales.

### Studies characteristics and risk of bias

Table 2 summarizes the main characteristics of the studies. Considering the psychometric properties evaluated, studies assessed hypothesis testing (41.63%) (i.e. concurrent and discriminative validity), reliability (test–retest) (16.66%), internal consistency (12.5%), error measurement (8.33%), criterion validity (4.16%), content validity (4.16%), instrument development (4.16%), cross‐cultural adaptation (4.16%), and structural validity (4.16%). Fifty per cent of the psychometric properties evaluated for all studies included were considered ‘very good’ according to the COSMIN Risk of Bias checklist, 29.16% as ‘adequate’, and 20.83% as ‘inadequate’. No properties were rated as ‘doubtful’.

**Table 2:** Characteristics of the studies.

Study	Instrument	Country	Population (age, functional classifications, ACSF)	Sample size	Psychometric properties evaluated	Risk of bias	Results	Quality criteria for measurement properties
Assessing the validity and reliability of the Chinese VABS for children with ASD aged 1–6 years^26^	VABS	China	1–6 years (functional classifications not reported)	2118 (230 for hypothesis testing)	Internal consistency Structural validity Hypotheses testing	Internal consistency: very good Structural validity: very good Hypotheses testing: very good	Internal consistency: *α* = 0.93–0.99 Structural validity: CFI = 0.90–0.99 Hypothesis testing (convergent validity): positive significant correlations with Gesell Developmental Schedules (*r* = 0.30–0.60, *p* < 0.05)	Internal consistency: sufficient (+) Structural validity: sufficient (+) Hypothesis testing (convergent validity): sufficient (+)
Validating motor delays across the DCDQ and VABS in children with ASD^27^	VABS and DCDQ	USA	1–10 years (functional classifications not reported)	2644	Hypotheses testing	Hypotheses testing: adequate	Hypothesis testing (concurrent validity): strong correlations between VABS and DCDQ scores (Pearson's correlation coefficient = 0.62)	Hypothesis testing (concurrent validity): sufficient (+)
Content validity of an instrument for motor assessment of young people with autism^28^	GMA‐AUT Checklist	Brazil	Not applicable	9	Content validity Instrument development	Content validity: inadequate Instrument development: inadequate	Content validity: content validity index: 0.88–1.00	Content validity: indeterminate (?) Instrument development: insufficient (−)
Getting into the game: evaluation of the reliability, validity, and utility of the Ignite Challenge scale for school‐age children with ASD^29^	Ignite Challenge and PEDI‐CAT	Canada	6–17 years; ACSF‐Social Communication level I or II	47	Reliability Measurement error Hypotheses testing	Reliability: adequate Measurement error: adequate Hypotheses testing: very good	Reliability: Interrater: ICC = 0.96 Intrarater: ICC = 0.91 Measurement error: MDC = 9.28 Hypothesis testing (concurrent validity): significant correlations between Ignite Challenge scores and PEDI‐CAT mobility domain (*r* = 0.54, *p* < 0.0001), and Social/Cognitive (*r* = 0.57, *p* < 0.0001) Hypothesis testing (discriminant validity): Ignite Challenge showed different results between age groups (best test score 59.4 [dp = 15.5] versus older child score 80.3 [dp = 10.1], *p* < 0,001), and also between ACSF‐Social Communication level (level I best score 73.8 [dp = 12.8] versus level II score 58.3 [dp = 20.9], *p* = 0.007).	Reliability: sufficient (+) Measurement error: indeterminate (?) Hypothesis testing (concurrent validity and discriminant validity): sufficient (+)
Reliability, validity and acceptability of the PEDI‐CAT with ASD scales for Australian children and young people on the autism spectrum^30^	PEDI‐CAT and VABS	Australia	3–18 years; ACSF‐Social Communication and all support levels included	134	Internal consistency Reliability Hypotheses testing	Internal consistency: very good Reliability: adequate Hypotheses testing: very good	Internal consistency: McDonald's Omega = 0.89–0.93 Reliability test–retest: ICC = 0.89–0.92 Hypothesis testing (convergent validity): positive significant correlations for the VABS (*r* = 0.51–0.74, *p* < 0.05)	Internal consistency: indeterminate (?) Reliability: sufficient (+) Hypothesis testing (convergent validity): sufficient (+)
Concurrent validity of two standardized measures of gross motor function in young children with ASD^31^	M‐FUN; PDMS‐2	USA	4–5 years Childhood Autism Rating Scale moderate (64%) and severe (36%)	22	Hypothesis testing	Hypothesis testing: adequate	Hypothesis testing: (concurrent validity): significant correlation between M‐FUN scale and gross motor scores of PDMS‐2 (*r* = 0.84, *p* < 0.05). Hypothesis testing (discriminant validity): strong correlation in the identification of children with medium and delayed motor skills (Cohen's *k* = 0.77 and *p* < 0.05)	Hypothesis testing: (concurrent validity and discriminant validity): sufficient (+)
The effect of visual supports on performance of the TGMD‐2 for children with ASD^32^	TGMD‐2	USA	3–10 years (functional classifications not reported)	22	Hypothesis testing (concurrent validity)	Hypothesis testing: very good	Hypothesis testing (concurrent validity): significant difference between groups with and without visual support, *p* = 0.003	Hypothesis testing: sufficient (+)
TGMD‐3 with the use of visual supports for children with ASD: validity and reliability^33^	TGMD‐3	USA	4–10 years (functional classifications not reported)	14	Internal consistency Reliability Hypothesis testing (concurrent validity)	Internal consistency: very good Reliability: very good Hypothesis testing: very good	Internal consistency: ASD group: α = 0.88 Locomotion 0.82 Ball skills 0.5 ASD + visual supports: α = 0.93 Locomotion0.93 Ball skills: 0.81 Reliability: ASD group: Test–retest: ICC = 0.91 Interrater: ICC = 0.98 Intrarater: ICC = 0.99 ASD + visual supports: Test–retest: ICC = 0.92 Interrater: ICC = 0.99 Intrarater: ICC = 0.99 Hypothesis testing (concurrent validity): the raw TGMD‐3 scores of children with ASD improved significantly using the TGMD‐3 visual support. (*p* = 0.01) Significant improvement using visual supports (*p* = 0.01).	Internal consistency with visual support: sufficient (+) Internal consistency without visual support: sufficient (+) Reliability with visual support: sufficient (+) Reliability without visual support: sufficient (+) Hypothesis testing (concurrent validity): sufficient (+)
Reliability and agreement of the TUG test in children and teenagers with ASD^34^	TUG	Spain	6–18 years (functional classifications not reported)	50	Reliability Measurement error	Reliability: adequate Measurement error: adequate	Reliability: Interrater: ICC = 0.99 Intrarater: ICC = 0.88 Error measurement: MDC = 0.06	Reliability: sufficient (+) Measurement error: indeterminate (?)
Evaluation of the DCDQ as a screening instrument for co‐occurring motor problems in children with ASD^35^	DCDQ and MABC‐2	Belgium	5–15 years (functional classifications not reported)	115	Internal consistency Criterion validity Hypotheses testing	Internal consistency: very good Criterion validity: very good Hypotheses testing: very good	Internal consistency: α = 0.91 Criterion validity: area under the curve = 0.720 Hypothesis testing (concurrent validity): strong correlations between DCDQ and MABC‐2 scores (Spearman's rank correlation coefficient = 0.60) Hypothesis testing (discriminant validity): significant correlations between ASD + DCD group versus ASD without DCD group. DCDQ total: *p* < 0.001 Control during movement: *p* < 0.001 Fine motor/writing: *p* < 0.001 General coordination: *p* < 0.001	Internal consistency: sufficient (+) Criterion validity: sufficient (+) Hypothesis testing (concurrent validity, discriminant validity): sufficient (+)
MABC‐2 transcultural adaptation and evaluation of children with ASD aged 7–10 years^36^	MABC‐2	Brazil	7–10 years (functional classifications not reported)	41	Cross‐cultural validity Hypotheses testing	Cross‐cultural validity: inadequate Hypotheses testing: inadequate	Cross‐cultural validity: not reported Hypothesis testing (convergent validity): positive correlations with the Raven's Colored Progressive Matrices test (*r* = 0.17–0.56, *p* < 0.05)	Cross‐cultural validity: indeterminate (?) Hypothesis testing (convergent validity): insufficient (−)

Abbreviations: ACSF, Autism Classification System of Functioning; ASD, autism spectrum disorder; CFI, comparative fit index; DCDQ, Developmental Coordination Questionnaire; GMA‐AUT, Gross Motor Assessment of Children and Adolescents with ASD; ICC, intraclass correlation coefficient; MABC‐2, Movement Assessment Battery for Children, Second Edition; MDC, minimum detectable change; M‐FUN, Miller Function and Participation Scales; PDMS‐2, Peabody Developmental Motor Scales, Second Edition; TGMD‐2, Test of Gross Motor Development, Second edition; TGMD‐3, Test of Gross Motor Development, Third edition; TUG, Timed Up and Go; VABS, Vineland Adaptive Behavior Scales.

Table 2 summarizes each risk of bias assessment for each measurement property evaluated in the selected studies. The complete risk of bias scoring with the criteria that lowered the risk of bias in the final score of each study and each measurement property is presented in Table S2.

### Quality of measurement properties and summary of results

Each measurement property index identified in the selected studies was classified into three categories: sufficient (+); insufficient (−); or indeterminate (?). About 69.56% of the properties of the measures presented sufficient (+) results, 21.73% presented indeterminate (?) results, and the rest (8.71%) presented insufficient (−) results. Within the sufficient results, 16.6% were reliability results, 33.3% were about hypothesis testing, 12% were about internal consistency, and 4% were about criterion validity. For the indeterminate (?) results, 8% were about measuring error; 4% were about internal consistency, 4% were about cross‐cultural adaptation, and the other 4% were about content validity. Among the insufficient (−) results, 4% were related to the development of the instrument and to hypothesis testing. The results for structural validity were sufficient (+).

### Level of evidence

**Figure 1:**
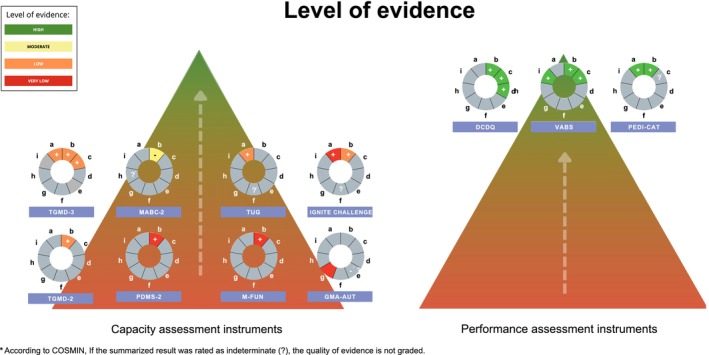
Representation of the level of evidence based on psychometric properties. GRADE level of evidence: green, high; yellow, moderate; orange, low; red, very low. a, reliability; b, hypothesis testing; c, internal consistency; d, criterion validity; e, content validity; f, error measurement; g, development of the instrument; h, cross‐cultural adaptation; i, structural validity; +, sufficient results; −, insufficient results; ?, undetermined results. Abbreviations: DCDQ, Developmental Coordination Disorder Questionnaire; GMA‐AUT, Gross Motor Assessment of Children and Adolescents with Autism Spectrum Disorder; MABC‐2, Movement Assessment Battery for Children, Second Edition; M‐FUN, Miller Function and Participation Scales; PDMS‐2, Peabody Developmental Motor Scales, Second Edition; PEDI‐CAT, Pediatric Evaluation of Disability Inventory Computer Adaptive Test; TGMD‐2, Test of Gross Motor Development, Second Edition; TGMD‐3, Test of Gross Motor Development, Third Edition; TUG, Timed Up and Go; VABS, Vineland Adaptive Behavior Scales.

Regarding the level of evidence, 39.13% of the properties evaluated were classified as having a high level of evidence, 4.34% as moderate, 34.78% as low, and the remaining 21.73% as very low. Figure 1 provides a representation of the level of evidence according to the number of properties evaluated, the GRADE classification, and the quality of the measurement properties. The figure represents the lowest level of evidence and insufficient (−) measurement property indices. At the same time, it shows the instruments with the highest level of evidence with sufficient (+) measurement properties. Thus, the instruments with the highest level of evidence and appropriate psychometric indices for the evaluated properties are shown at the top. The detailed levels of evidence classification, according to the GRADE criteria, are provided in Table S3.

#### Reliability

Among the included studies, five investigated the reliability of four instruments: Ignite Challenge,^29^ PEDI‐CAT,^30^ TGMD‐3,^33^ and TUG.^34^ The TGMD‐3 and TUG were classified as having a low level of evidence because of very serious imprecision in the evaluation of the TGMD‐3 and very serious risk of bias associated with serious imprecision. The Ignite Challenge was classified as having a very low level of evidence because of serious risk of bias and serious imprecision. However, the PEDI‐CAT was classified as having a high level of evidence because it presented no risk of bias and had consistent results.

#### Hypothesis testing

Nine instruments evaluated hypothesis testing as a measurement property: the VABS,^26^, ^27^ Ignite Challenge,^29^ PEDI‐CAT,^30^ M‐FUN, PDMS‐2,^31^ TGMD‐2,^32^ TGMD‐3,^33^ DCDQ,^35^ and MABC‐2.^36^ The TGMD‐2, TGMD‐3, and Ignite Challenge were classified as having a low level of evidence because of very serious imprecision. The M‐FUN and PDMS‐2 were classified as having a very low level of evidence because of serious risk of bias and very serious imprecision. The DCDQ, VABS, and PEDI‐CAT were classified as having a high level of evidence, supported by consistent results. The MABC‐2 was classified as having a low level of evidence because of serious imprecision and risk of bias.

#### Internal consistency

The internal consistency of the VABS,^26^ TGMD‐3,^33^ and DCDQ^35^ was evaluated. A high level of evidence was assigned to the DCDQ and VABS because of consistent results and the absence of risk of bias. However, the TGMD‐3 was classified as having a low level of evidence because of very serious imprecision. The PEDI‐CAT^30^ presented indeterminate internal consistency (?) with no level of evidence thus far.

#### Criterion validity

The DCDQ^35^ was classified as having a high level of evidence because of the absence of risk of bias and because of consistent results.

#### Content validity

The GMA‐AUT^28^ presented indeterminate content validity (?); therefore no level of evidence was graded.

#### Measurement error

In two instruments, Ignite Challenge^29^ and TUG,^34^ measurement error was evaluated. Both presented no level of evidence rated because of indeterminate (?) measurement property quality.

#### Instrument development

The GMA‐AUT^28^ was classified as having a very low level of evidence because of an extremely serious risk of bias and serious imprecision.

#### Cross‐cultural adaptation

The MABC‐2^36^ was classified as having a low level of evidence because of serious imprecision and risk of bias.

#### Structural validity

The VABS^26^ was classified as having a high level of evidence because of no risk of bias and because of consistent results.

### Clinical decision map based on the level of evidence

**Figure 2:**
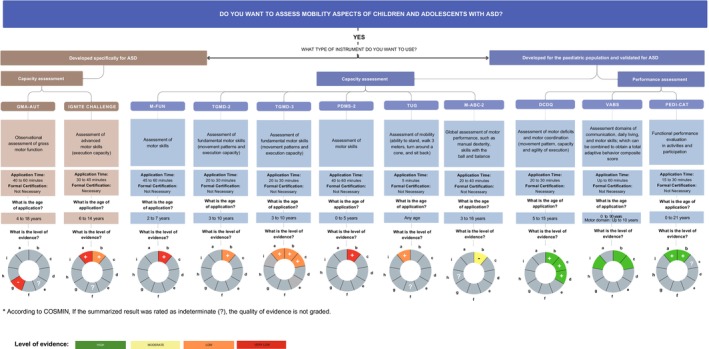
Decision map tree. a, reliability; b, hypothesis testing; c, internal consistency; d, criterion validity; e, content validity; f, error measurement; g, development of the instrument; h, cross‐cultural adaptation; i, structural validity; +, sufficient results; −, insufficient results;?, undetermined results. Abbreviations: ASD, autism spectrum disorder; DCDQ, Developmental Coordination Disorder Questionnaire; GMA‐AUT, Gross Motor Assessment of Children and Adolescents with Autism Spectrum Disorder; MABC‐2, Movement Assessment Battery for Children, Second Edition; M‐FUN, Miller Function and Participation Scales; PDMS‐2, Peabody Developmental Motor Scales, Second Edition; PEDI‐CAT, Pediatric Evaluation of Disability Inventory Computer Adaptive Test; TGMD‐2, Test of Gross Motor Development, Second Edition; TGMD‐3, Test of Gross Motor Development, Third Edition; TUG, Timed Up and Go; VABS, Vineland Adaptive Behavior Scales.

The clinical decision map summarizes the data found in this review, pointing out the variable aspects included in the instruments' items and the age range covered by each of them (Figure 2).

## DISCUSSION

This systematic review aimed to investigate the instruments available for assessing mobility aspects and analyse the quality and level of evidence of their measurement properties in the population with ASD. Nine instruments developed for typically developing children and validated for ASD were identified, along with two instruments specifically developed for ASD. Positive and high evidence was found for the DCDQ (hypothesis testing, internal consistency, and criterion validity), VABS (hypothesis testing, internal consistency, and structural validity), and PEDI‐CAT (reliability and hypothesis testing). Conversely, positive low evidence was found for Ignite Challenge (hypothesis testing), TGMD‐2 (hypothesis testing), TGMD‐3 (reliability, hypothesis testing, and internal consistency), TUG (reliability), and MABC‐2 (hypothesis testing and cross‐cultural adaptation). Positive very low evidence was identified for Ignite Challenge (reliability), PDMS‐2 (hypothesis testing), and M‐FUN (hypothesis testing). GMA‐AUT presented negative low and indeterminate evidence for content validity and instrument development. The results show that there is higher evidence regarding performance tools compared with capacity tools.

The classifications in this study reveal the quality and level of evidence of instruments designed to assess the functional features of mobility aspects. These instruments evaluate different constructs of mobility aspects within performance and capacity. It is worth noting that none of the studies evaluated all measurement properties deemed important by COSMIN.^23^ Each reviewed instrument presents distinct advantages and limitations, particularly regarding the breadth of evaluated properties and their applicability in clinical practice. Based on this review's findings, the DCDQ, VABS, and PEDI‐CAT are the instruments with the highest level of evidence for assessing mobility aspects in children and adolescents with ASD. Additionally, the VABS and PEDI‐CAT stand out for their comprehensiveness in evaluating other relevant aspects of functionality, such as daily activities, communication, and social responsibilities.^26^, ^27^, ^30^ The results for these instruments were generally consistent and sufficient for investigating reliability, hypothesis testing, criterion validity, and structural validity, ensuring their use in clinical practice.

The instruments assessing capacity, that is, the GMA‐AUT, Ignite Challenge, PDMS‐2, M‐FUN, TGMD‐2, TGMD‐3, TUG, and MABC‐2 showed moderate to very low levels of evidence for their evaluated properties. Imprecision and risk of bias were key factors affecting their evidence. Risk of bias was determined according to the evaluation criteria for each assessed property.^23^ Imprecision was based on the sample size included in each study.^25^ Sample sizes for children and adolescents with ASD were notably smaller for capacity instruments compared with performance questionnaires. This may reflect the relative ease of administering standardized parent questionnaires versus conducting in‐person observational evaluations required for capacity tests. Consequently, performance questionnaires tend to have higher levels of evidence than capacity tests in children with ASD. Future studies should prioritize evaluating capacity instruments with adequate sample sizes to reduce imprecision.

Instruments such as the VABS, PEDI‐CAT, PDMS‐2, TGMD, and MABC‐2 are widely studied in different neurodevelopmental disorders. To the best of our knowledge, there is no systematic review that evaluates the quality of their psychometric properties, according to the COSMIN standards, when considering the general population with disabilities. However, we recognize that these instruments may demonstrate different levels of evidence regarding the quality of their measurement properties when considering populations with neurodevelopmental disorders more broadly. To provide more specific interpretations of the level of evidence for their use in children and adolescents with ASD, future studies with larger sample sizes and stronger methodological quality are needed to strengthen the evidence base for these instruments.

Moreover, investigation of the properties of the Ignite Challenge and M‐FUN instruments lacks studies that better assess their applicability and validity. To date, few studies have been dedicated to this investigation. In the study by Wright et al.,^29^ the reliability and hypothesis testing properties evaluated showed sufficient results. As in the study by Wright et al.,^29^ the hypothesis testing evaluated by Holloway et al.^31^ also yielded consistent results. With more in‐depth investigations in the future, the level of evidence for these instruments may change. Future methodological studies focused on investigating assessment instruments for ASD should ensure that the specific methodological aspects related to the properties being examined and the sample size are adequate and have sufficient statistical power to support the quality of the evidence being investigated.

Reliability is an important property to consider.^24^, ^37^ Only 5 of 11 instruments assessed this measurement property. Reliability refers to the degree to which patients can be differentiated from one another, considering measurement errors.^37^ For example, no studies investigated the reliability of TGMD‐2 in samples of children with ASD, only comparing a small portion of children with disabilities to typically developing children. Given the variability of motor impairments in ASD, the importance of a study specifically investigating these normative data for ASD is evident. These findings are aligned to data presented in the review by Wilson et al.,^17^ which shows that the greatest limitation is related to the absence of children and adolescents with ASD in the normative samples of the instruments.

Like reliability, responsiveness is a property that should be investigated. According to Terwee et al.,^37^ responsiveness defines an instrument's ability to detect clinically important changes over time, even small ones. None of the 11 studies included in this review evaluated instrument responsiveness. Assessing responsiveness is fundamental because it determines the instrument's ability to detect significant changes over time, especially in clinical intervention contexts.^22^, ^37^ For children and adolescents with ASD, this analysis is particularly relevant because it helps identify whether the observed changes reflect the tangible effects of an intervention. Studies evaluating responsiveness contribute to scientific advancement, ensuring that instruments are not only valid and reliable but also responsive to changes critical to the functionality of individuals with ASD.

Another important property investigated only in one study is cross‐cultural adaptation. This property concerns the extent to which items in a translated or culturally adapted instrument appropriately reflect the original instrument's items.^24^ Cross‐cultural adaptation ensures that the psychometric properties of the original instrument are preserved in the adapted version, thus ensuring that it continues to measure its intended constructs across cultural contexts. Some of the instruments included, such as the PEDI‐CAT and TGMD‐2, are widely cross‐culturally translated; however, as the cross‐cultural translation process did not include individuals with ASD, these studies were not included in this review.^38^, ^39^

It is important to highlight that the evaluation of mobility aspects in children with ASD might be complemented by assessing other body functions and structures. After evaluating performance and capacity limitations in children and adolescents with ASD, therapists should investigate what motor components (e.g. balance, coordination, strength) might hinder children's mobility. For that purpose, popular instruments such as the Bruininks–Oseretsky Test of Motor Proficiency might be useful.^40^, ^41^

### Limitations and future directions

The review eligibility criterion deliberately required the inclusion of studies that investigated measurement properties in samples predominantly consisting of children and adolescents with ASD to ensure that the psychometric properties analysed were specifically directed to this population. This decision was essential to preserve the clinical relevance and validity of the findings because including methodological studies in which less than 50% of participants had ASD could lead to serious indirectness and compromise the accuracy of the evidence level. By maintaining this focus, the review provides evidence‐based information representative of the population with ASD, which supports more precise and context‐appropriate clinical decision‐making. However, other tools developed for populations with broader neurodevelopmental disorders might still be useful alternatives when the instruments included in this review do not fully meet therapists' purposes. In such cases, the selection of the most suitable assessment tool should always be guided by the specific needs and goals of the child and family, and by the individual characteristics of the child being evaluated.

Variability in the instruments' content (i.e. inclusion of different mobility aspects) and the diverse age ranges of participants hindered more in‐depth comparisons beyond capacity and performance. The choice of an instrument should primarily consider the individual's characteristics, goals, and context. The clinical decision map presented in this study may assist clinicians in selecting the most appropriate mobility instruments for different situations. Considering the challenges of evaluating children with ASD, especially in capacity tests, future studies should include larger samples of children and adolescents with ASD to establish levels of evidence specific to this population. Also, the lack of information regarding ASD functional classifications and diagnosis methods in the studies included in this review limited the possibility of conducting a deeper and more nuanced analysis of the results. Moreover, future research should investigate properties that have not yet been evaluated, such as responsiveness.

## Conclusions

This review investigated the quality and level of evidence of the measurement properties of nine instruments used to assess functional mobility aspects. Most of the measurement properties of the instruments demonstrated low or very low evidence because of risk of bias and imprecision. The DCDQ, VABS, and PEDI‐CAT were identified as the instruments with the highest level of recommendation regarding their evidence for evaluating mobility aspects in children and adolescents with ASD. On the other hand, the Ignite Challenge, PDMS‐2, M‐FUN, GMA‐AUT, TGMD‐2, TGMD‐3, TUG, and MABC‐2 presented moderate to very low levels of evidence for the properties being evaluated. The findings highlighted the need for future analyses of the measurement properties of instruments for assessing mobility aspects according to the COSMIN guidelines.

## Supporting information


**Figure S1:** Flowchart.

**Table S1:** Search strategy conducted in October 2023

**Table S2:** Summarized results according to the Risk of Bias Checklist

**Table S3:** Summarized results according to the measurement properties

## Data Availability

The data that support the findings of this study are available from the corresponding author upon reasonable request.
