# Supplementary material for: Instruments assessing mobility of children and adolescents with autism spectrum disorder: A systematic review and decision map
Source: Dev Med Child Neurol. 2025 Dec 29;68(8):1049–62. doi: 10.1111/dmcn.70136 (PMC13340621; doi:10.1111/dmcn.70136)
Supplement: Supplementary file 1 — Figure S1: Flowchart. [file DMCN-68-1049-s004.docx]

Additional records identified through handsearching: (n= 10)

It did not evaluate samples mostly composed of individuals with ASD (9);

Did not assess motor outcome (2);

Abstract (1);

Screening Instrument (2)

**Screening**


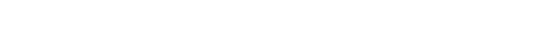


**Eligibility**

Records identified through database setting:

(n = 6098)

Duplicates removed

(n = 1397)

Records screened

(

n

=4711)

Excluded records after abstract

reading

=4686)

n

(

Full-text articles assessed for eligibility (n=25)

Full-text articles excluded: (n=14)

Studies included in synthesis

(n = 11)

**Included**


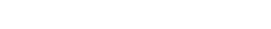


**Identification**


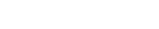


Figure S1. Flowchart.
