# Supplementary material for: Instruments assessing mobility of children and adolescents with autism spectrum disorder: A systematic review and decision map
Source: Dev Med Child Neurol. 2025 Dec 29;68(8):1049–62. doi: 10.1111/dmcn.70136 (PMC13340621; doi:10.1111/dmcn.70136)
Supplement: Supplementary file 3 — Table S2: Summarized results according to the Risk of Bias Checklist [file DMCN-68-1049-s002.docx]

**Table S2**. Summarized results according to the Risk of Bias Checklist.

| **Instrument** | **Measurement Properties** | **Risk of bias score** | **Criteria that lowered the risk of bias final score** |
| --- | --- | --- | --- |
| **Gross Motor Assessment of Children and Adolescents with Autism Spectrum Disorder (GMA-AUT)**  Heidrich et al., 2018 | Content Validity | Inadequate | Only quantitative (survey) method(s) used or assumable that the method was appropriate but not clearly described and assumable that the approach was appropriate, but not clearly described |
|  | Instrument Development: | Inadequate | Method used not appropriate or not suitable for the construct or study population |
| **Ignite Challenge**  Wright et al., 2023 | Reliability | Adequate | Assumable that patients were stable |
|  | Hypotheses Testing | Very good | Not applicable |
|  | Measurement error | Adequate | Assumable that patients were stable |
| **Miller Function and Participation Scales (M-FUN) and Peabody Developmental Motor Scales, Second Edition (PDMS-2)**  Holloway et al., 2019 | Hypotheses Testing | Adequate | Sufficient measurement properties of the comparator instrument(s) but not sure if they apply to the study population |
| **Test of Gross Motor Development (TGMD-2)**  Breslin et al., 2011 | Hypotheses Testing | Very good | Not applicable |
| **Test of Gross Motor Development (TGMD-3)**  Allen et al., 2017 | Reliability | Very good | Not applicable |
|  | Hypotheses Testing | Very good | Not applicable |
|  | Internal Consistency | Very good | Not applicable |
| **Peabody Developmental Motor Scales, Second Edition (PDMS-2)**  Holloway et al., 2019 | Hypotheses Testing | Adequate | Sufficient measurement properties of the comparator instrument(s) but not sure if these apply to the study population |
| **Timed Up and Go (TUG)**  Martin-Diaz et al., 2023 | Reliability | Adequate | Assumable that patients were stable |
|  | Measurement error | Adequate | Assumable that patients were stable |
| **Movement Assessment Battery for Children-2 (MABC-2)**  Quedas et al., 2021 | Hypotheses Testing | Inadequate | Constructs measured by the comparator instrument(s) is not clear |
|  | Cross-Cultural Adaptation | Inadequate | Methods not appropriate |
| **Developmental Coordination Questionnaire (DCDQ)**  Van Damme et al., 2022 | Hypotheses Testing | Very good | Not applicable |
|  | Internal Consistency | Very good | Not applicable |
|  | Criterion Validity | Very good | Not applicable |
| **Vineland Adaptive Behavior Scales (VABS)**  Deng et al., 2025; Bhat et al., 2024 | Hypotheses Testing | Very good | Not applicable |
|  | Internal Consistency | Very good | Not applicable |
|  | Structural Validity | Very good | Not applicable |
|  | Hypotheses Testing | Adequate | Assumable that statistical method was appropriate |
| **Pediatric Evaluation of Disability Inventory-Computer Adaptive Test for autism (PEDI-CAT)**  Chamberlain et al., 2024; Wright et al., 2023 | Reliability | Adequate | Assumable that patients were stable |
|  | Hypotheses Testing | Very good | Not applicable |
|  | Internal Consistency | Very good | Not applicable |
