# Supplementary material for: Instruments assessing mobility of children and adolescents with autism spectrum disorder: A systematic review and decision map
Source: Dev Med Child Neurol. 2025 Dec 29;68(8):1049–62. doi: 10.1111/dmcn.70136 (PMC13340621; doi:10.1111/dmcn.70136)
Supplement: Supplementary file 4 — Table S3: Summarized results according to the measurement properties [file DMCN-68-1049-s003.docx]

**Table S3**. Summarized results according to the measurement properties.

| **Reliability** | **Summarized results** | **Total sample** | **General evaluation** | **Level of evidence (GRADE)** |
| --- | --- | --- | --- | --- |
| **Test of Gross Motor Development (TGMD-3)**  Allen et al., (2017) | ICC= ranging between 0.91-0.99, | n=14 | Sufficient (+) | **Low level of evidence** (very serious imprecision) |
| **Timed Up and Go (TUG)**  Martín-Díaz et al., (2023) | ICC= ranging between 0.88-0.99 | n= 50 | Sufficient (+) | **Low level of evidence** (very serious risk of bias and serious imprecision) |
| **Ignite Challenge**  Wright et al., 2023 | ICC= ranging between 0.91-0.96 | n=47 | Sufficient (+) | **Very low level of evidence** (serious risk of bias and very serious imprecision) |
| **Pediatric Evaluation of Disability Inventory - Computer Adaptive Test (PEDI-CAT)**  Chamberlain et al., 2024 | ICC= ranging between 0.89-0.92 | n=134 | Sufficient (+) | **High level of evidence** (no risk of bias and consistent results) |

| **Hypothesis Testing** | | **Summarized results** | **Total sample** | **General evaluation** | **Level of evidence (GRADE)** |
| --- | --- | --- | --- | --- | --- |
| **Test of Gross Motor Development (TGMD-2)**  Breslin et al., 2011 | | Significant difference between groups with and without visual support p=0.003 | n=22 | Sufficient (+) | **Low level of evidence** (very serious imprecision) |
| **Test of Gross Motor Development (TGMD-3)**  Allen et al., 2017 | | The raw TGMD-3 scores of children with ASD improved significantly using the TGMD-3 visual support. (p=0.01). Significant improvement using visual supports (p=0.01). | n=15 | Sufficient (+) | **Low level of evidence** (very serious imprecision) |
| **Miller Function and Participation Scales (M-FUN)**  Holloway et al., 2019 | | Concurrent validity: Significant correlation between M-FUN scale and gross motor scores of PDMS-2(r= 0.84, p<0.05).  Discriminant validity:  Strong correlation in identification of children with medium and delayed motor skills (Cohen´s k=0.77 and p<0.05). | n=22 | Sufficient (+) | **Very low level of evidence** (serious risk of bias and very serious imprecision) |
| **Peabody Developmental Motor Scales, Second Edition (PDMS-2)**  Holloway et al., 2019 | |  |  |  |  |
| **Ignite Challenge**  Wright et al., 2023 | | Concurrent validity:  Significant correlations between Ignite Challenge scores and PEDI-CAT mobility domain (r= 0.54, p <0.0001), and  Social/Cognitive (r = 0.57, p<0.0001)  Discriminant validity: Ignite Challenge show different results between age groups (best test score 59.4 (dp=15.5) versus older child score 80.3 (dp= 10,1), p < 0,001), and also between ACSF:SC level (Level I best score 73.8 (dp= 12.8) versus level II score 58.3 (dp=20.9), p= 0.007). | n= 47 | Sufficient (+) | **Low level of evidence** (very serious imprecision) |
| **Developmental Coordination Questionnaire (DCDQ)**  Van Damme et al., 2022 | | Concurrent validity:  Strong correlations between DCDQ scores and M-ABC scores (spearman = 0.60)  Discriminant validity: Significant correlations between ASD + DCD group versus ASD without DCD group.  DCDQ total: p<.001  - Control during movement: p <.001  - Fine Motor/Writing: p <.001  - General coordination: p <.001 | n=115 | Sufficient (+) | **High level of evidence** (no risk of bias and consistent results) |
| **Pediatric Evaluation of Disability Inventory-Computer Adaptive Test for autism (PEDI-CAT)**  Chamberlain et al., 2024 | | Convergent validity:  Positive significant correlations VABS (r=0.51-0.74, p<0.05) | N=134 | Sufficient (+) | **High level of evidence** (no risk of bias and consistent results) |
| **Movement Assessment Battery for Children-2 (MABC-2)**  Quedas et al., 2021  Van Damme et al., 2022 | | Convergent validity:  Positive significant correlations with Gessel Developmental Schedules (r=0.30-0.60, p<0.05) | n=156 | Insufficient (-) | **Moderate level of evidence** (serious risk of bias) |
| **Vineland Adaptive Behavior Scales (VABS)** | Deng et al., 2025 | Concurrent validity: Significant correlations were observed between the CVABS-III and the Gesell Developmental Schedules (GDS), particularly in the 3–6 age group, with strong associations across communication (r=0.79, r=0.79), daily living skills (r=0.44–0.53, r=0.44–0.53), and socialization domains (r=0.47–0.53,  r=0.47–0.53), all p<0.001, p<0.001.  Discriminant validity: The CVABS-III effectively differentiated between typically developing children and those with ASD or DD across all age groups, with large effect sizes (Cohen’s d>0.7 d>0.7 in multiple domains for ages 3–6, p<0.001 p<0.001), and showed significant but smaller differences for children with SLDD. | n=2713 | Sufficient (+) | **High level of evidence** (no risk of bias and consistent results) |
|  | Bhat et al., 2024 | Concurrent validity: Significant correlation between the DCD-Q and the Vineland Adaptive Behavior Scales (VABS) motor domain scores (r=0.62r=0.62, p<0.0001  p<0.0001).  Discriminant validity: High accuracy (81.2%) in identifying concurrent motor delays between the DCD-Q and VABS, with a positive predictive value of 70.4%. |  |  |  |

| **Internal Consistency** | **Summarized results** | **Total sample** | **General evaluation** | **Level of evidence (GRADE)** |
| --- | --- | --- | --- | --- |
| **Test of Gross Motor Development (TGMD-3)**  Allen et al., 2017 | With visual support: Cronbach's α of 0.93;  No visual support: Cronbach's α of 0.88;. | n=14. | Sufficient (+) | **Low level of evidence** (very serious imprecision) |
| **Developmental Coordination Questionnaire (DCDQ)**  Van Damme et al., 2022 | Cronbach's α of 0.91; | n=115 | Sufficient (+) | **High level of evidence** (no risk of bias and consistent results) |
| **Pediatric Evaluation of Disability Inventory-Computer Adaptive Test for autism (PEDI-CAT)**  Chamberlain et al., 2024 | McDonald Omega between 0.89 - 0.93; | N=134 | Indeterminate (?) | **High level of evidence** (no risk of bias and consistent results) |
| **Vineland Adaptive Behavior Scales (VABS)**  Deng et al., 2025 | Cronbach's α of 0.93-0.99 | n=2252 | Sufficient (+) | **High level of evidence** (no risk of bias and consistent results) |

| **Criterion Validity** | **Summarized results** | **Total sample** | **General evaluation** | **Level of evidence (GRADE)** |
| --- | --- | --- | --- | --- |
| **Developmental Coordination Questionnaire (DCDQ)**  Van Damme et al., 2022 | Area under the curve 0.72 | n=115 | Sufficient (+) | **High level of evidence** (no risk of bias and consistent results) |

| **Content Validity** | **Summarized results** | **Total sample** | **General evaluation** | **Level of evidence (GRADE)** |
| --- | --- | --- | --- | --- |
| **Gross Motor Assessment of Children and Adolescents with ASD**  Heidrich et al., 2022 | CVI* between 0.88 a 1.00  *Content Validity Index (CVI) | n= 8 | Indeterminate (?) | **Very low level of evidence** (extremely serious risk of bias) |

| **Error Measurement** | **Summarized results** | **Total sample** | **General evaluation** | **Level of evidence (GRADE)** |
| --- | --- | --- | --- | --- |
| **Timed Up and Go (TUG)**  Martin-Diaz et al., 2023 | Standard error of measurement of 0.02; with minimum detectable change of 0.06. | n= 50 | Indeterminate (?) | **Low level of evidence** (serious risk of bias and serious imprecision) |
| **Ignite Challenge**  Wright et al., 2023 | Standard measurement error of 5.13; with minimum detectable change of 9.28. | n= 47 | Indeterminate (?) | **Very low level of evidence** (serious risk of bias and very serious imprecision) |

| **Instrument Development** | **Summarized results** | **Total sample** | **General evaluation** | **Level of evidence (GRADE)** |
| --- | --- | --- | --- | --- |
| **Gross Motor Assessment of Children and Adolescents with ASD**  Heidrich et al., 2022 | In the final version of the instrument, only two items presented  CVI* of 0.88, while all the others had CVI* of 1.00.  *Content Validity Index (CVI) | n= 8 | Insufficient (-) | **Very low level of evidence** (extremely serious risk of bias and very serious imprecision) |

| **Cross-cultural adaptation** | **Summarized results** | **Total sample** | **General evaluation** | **Level of evidence (GRADE)** |
| --- | --- | --- | --- | --- |
| **Movement Assessment Battery for Children-2 (MABC-2)**  Quedas et al., 2021 | It was observed that the correlations with the standard scores between the domains assessed by MABC-2 and the CPM results showed to be median Manual Dexterity (r = 0.454, p = 0.012) and Balance (r = 0.324, p = 0.081) and small for Aiming and Catching (r = 0.170, p = 0.368). | n=41 | Indeterminate (?) | **Low level of evidence** (serious risk of bias and serious inconsistency) |

| **Structural Validity** | **Summarized results** | **Total sample** | **General evaluation** | **Level of evidence (GRADE)** |
| --- | --- | --- | --- | --- |
| **Vineland Adaptive Behavior Scales (VABS)**  Deng et al., 2025 | CFI=0.90-0.99 | N=2252 | Sufficient (+) | **High level of evidence** (no risk of bias and consistent results) |
